# BSA-Silver Nanoparticles: A Potential Multimodal Therapeutics for Conventional and Photothermal Treatment of Skin Cancer

**DOI:** 10.3390/pharmaceutics13040575

**Published:** 2021-04-17

**Authors:** Dasom Kim, Reeju Amatya, Seungmi Hwang, Sumi Lee, Kyoung Ah Min, Meong Cheol Shin

**Affiliations:** 1 College of Pharmacy and Research Institute of Pharmaceutical Sciences, Gyeongsang National University, 501 Jinju Daero, Jinju, Gyeongnam 52828, Korea; ekthadk2004@naver.com (D.K.); reejuamatya94@gmail.com (R.A.); 2 College of Pharmacy and Inje Institute of Pharmaceutical Sciences and Research, Inje University, 197 Injero, Gimhae, Gyeongnam 50834, Korea; hsm8549@naver.com (S.H.); dltnal333@naver.com (S.L.)

**Keywords:** silver nanoparticle, bovine serum albumin, photothermal therapy, angiogenesis, skin cancer

## Abstract

Silver nanoparticles (NPs) have attracted a considerable interest in the field of cancer research due to their potential utility in cancer therapy. In the present study, we developed bovine serum albumin (BSA)-coated silver NPs (BSA-Silver NPs) and characterized in vitro multimodal therapeutic activities of NPs for the treatment of skin cancer. BSA-Silver NPs were synthesized by a single-step reduction process, and the successful preparation was verified through a list of physical characterizations, including transmission electron microscopy (TEM), ultraviolet-visible (UV–VIS) light spectroscopy, dynamic light scattering (DLS), and Fourier transform infrared (FT-IR) spectroscopy. The synthesized BSA-Silver NPs showed marked cytocidal effects on B16F10 melanoma cells, which was likely caused by oxidative stress. BSA-Silver NPs also elicited significant anti-angiogenic effects on HUVEC (human umbilical vein endothelial cell) by inhibiting their proliferation, migration, and tube formation. Moreover, BSA-Silver NPs showed a considerable light-to-heat conversion ability, suggesting their utility as photothermal agents. Overall, our findings suggest that BSA-Silver NPs may be promising candidates for the multimodal therapy of skin cancer.

## 1. Introduction

In recent years, silver nanoparticles (NPs) have gained great attention for their utility in various fields, including cancer therapy [1,2]. Silver NPs have been reported to be highly cytotoxic to various cancer cell lines [3]. There is a broad consensus that the main mechanism for this cytotoxicity is related to the oxidative stress-mediated by reactive oxygen species (ROS) [4,5]. Furthermore, in a recent study, Yang et al. reported that silver NPs could also actuate cytotoxicity by disrupting the HIF signaling pathway [6]. Whether there is a difference in the sensitivity of the silver NPs’ action on cancer cells over normal cells remains controversial, as the results of previous reports are inconsistent [2,7]. Yet, there have been accumulating research efforts to develop more effective, but safer ways to exploit the silver NPs’ activity in cancer treatment [8,9].

Tumor angiogenesis supports the oxygen supply and essential nutrients for tumor growth as well as allows for metastasis of tumor cells to distant sites [10,11]. Accordingly, tumor angiogenesis has been one of the primary targets in cancer treatment and, indeed, many anti-angiogenic drugs have been discovered and introduced to clinical practice [12]. Interestingly, certain NPs including chitosan, silica, selenium, gold, and silver NPs also possess the activity to inhibit the angiogenesis and could potentially serve as effective anti-angiogenic agents [13,14]. The mechanisms for the antiangiogenic activity of these NPs depends on diverse pathways, including direct interaction with molecular targets (e.g., VEGF165, bFGF, etc.), downregulation of VEGFR2 and suppression of FGFR1, Erk1/2, Akt, and VEGFR2 phosphorylation [13]. There is evidence that the types of coating material and the size of the NPs could affect their inhibitory activity, specifically if the mechanism requires the NPs to internalize and interact with target molecules [15].

Photothermal therapy (PTT) is a mode of hyperthermic treatment that uses light-to-heat converting materials as the heat source [16]. Either alone or combined with conventional chemo/radiotherapy, hyperthermia is known to provide various therapeutic benefits for the treatment of cancer [17,18]. As for combination therapy, hyperthermia could radiosensitize cancer cells and increase the perfusion in tumors, leading to a higher delivery of anti-cancer drugs [19]. On the other hand, it could also directly ablate cancer cells. Previous reports suggested that temperatures around 43–50 °C could induce cancer cell death with some extent of selectivity [20]. Gold NPs have gained the earliest interest as PTT agents, which was due to these NPs’ well-known ability for the absorption of near-infrared (NIR) light and great light-to-heat energy conversion efficiency [21]. However, soon afterwards, there have been accumulating reports of other NPs (e.g., gold, iron oxide, copper sulfide, and manganese oxide nanoparticles) that were found capable of converting light to heat [22,23]. To date, other than the discovery of various PTT agents, there have also been considerable advancements in their way of application in the cancer therapy. PTT has been found capable of not only directly ablating tumor cells, but also enhancing the effects of chemotherapy, photodynamic therapy, and immunotherapy [24]. Specifically, PTT-combined immunotherapy has recently been a hot topic, due to its potent immuno-stimulatory activity for promoting dendritic cell maturation. Although not as popular as other PTT agents, silver NPs have also been reported to possess this photo-thermal converting activity, which renders them potentially effective photothermal cancer therapeutic agents [25,26,27,28]. Indeed, there have been increasing reports demonstrating the applicability of silver NP-based PTT for anti-microbial and anti-cancer treatments [25,27]. 

In the present study, we developed BSA-Silver NPs and characterized their physicochemical properties by using TEM, UV-VIS spectrum analysis, DLS, and FT-IR. Then, the multimodal activities of BSA-Silver NPs for cancer therapy were explored in vitro focusing on their cytocidal, anti-angiogenic, and photothermal activity. The results of the present study suggest that BSA-Silver NPs could serve as potentially effective agents for the treatment of skin cancer.

## 2. Materials and Methods

### 2.1. Materials

Sodium borohydride (NaBH_4_) and silver nitrate (AgNO_3_) were purchased from Sigma-Aldrich (St. Louis. MO, USA). BSA was purchased from Amresco (Solon, OH, USA). Medium 200 and LSGS (low serum growth supplement) were obtained from ThermoFisher Scientific (Waltham, MA, USA). All other chemicals were of the analytical grade.

### 2.2. Synthesis of BSA-Silver NPs

First, 100 mg of BSA were dissolved in 30 mL of double-distilled water (DDW), and then 1 mg of NaBH_4_ was added as the reducing agent. While stirring at 1000 rpm under room temperature (RT), 4 mL of 10^−2^ M silver nitrate (AgNO_3_) solution were drop-by-drop added to the BSA/NaBH_4_ solution with a 30-gauge needle syringe and incubated for 30 min. After incubation, the NaBH_4_ and non-incorporated BSA were removed using an ultracentrifugal device (membrane cut-off: 100 kDa; Amicon^®^ Ultra-15 centrifugal filter units, Merck Millipore, Darmstadt, Germany). Briefly, the reaction mixture was added to the ultracentrifugal device and centrifuged at 3000 rpm for 10 min. After the first run, DDW was added to the device and centrifuged under the same condition for 3 more times. The final BSA-Silver NP samples were prepared at 4 × 10^−2^ M (as silver) concentration and kept in the refrigerator (4 °C) until further use. Prior to cellular studies, the BSA-Silver NP suspension was filtered through a 0.45-μm-pore-size syringe filter.

### 2.3. Physical Characterization of BSA-Silver NPs

The size and morphology of the BSA-Silver NPs were examined using TEM (Tecnai 12, FEI, Hillsboro, OR, USA). The hydrodynamic size and zeta potential of the nanoparticles were measured by DLS (Zetasizer Nano ZS, Malvern Panalytical Ltd., Malvern, UK). To verify the formation of BSA-Silver NPs, the UV-VIS absorption spectra of the samples (at low, medium, and high concentration) were detected in the range of 300–800 nm with a Synergy H1 Hybrid Multi-Mode Reader (BioTek U.S., Winooski, VT, USA). Next, to identify the incorporation of BSA into the NPs, FT-IR spectrophotometry was performed (VERTEX 80v, Bruker, Billerica, MA, USA). The BSA and silver contents were quantified by the BCA assay and inductively coupled plasma mass spectrometry (ICP-MS), respectively.

### 2.4. Cell Culture

B16F10 murine melanoma cells were purchased from Korean cell line bank (KCLB, Seoul, Korea). HUVEC (human umbilical vein endothelial cells) were a gift from Prof. Jung-Hwan Kim (Gyeongsang National University, Korea). Both cell lines were cultured in a complete DMEM medium containing 10% FBS, 1% antibiotic antimycotic (AA), and 1% penicillin-streptomycin (PS), and the culture was maintained in a humidified incubator (5% CO_2_) at 37 °C.

### 2.5. Cytotoxicity Assay

B16F10 cells were seeded onto 96-well plates (5 × 10^3^ cells/well) and incubated overnight (o.n.). The cells were then treated with either silver ions (AgNO_3_) or BSA-Silver NPs (at 10^−8^–10^−2^ M as silver). After incubation for 48 h, the relative cell viability was determined using the WST-1 assay according to the manufacturer’s protocol (iNtRON Biotechnology, Daejeon, Korea). 

### 2.6. Detection of ROS Generation

The B16F10 cells were seeded onto 96-well plates (2.5 × 10^4^ cells/well). After o.n. incubation, the BSA-Silver NPs (0, 2.5 × 10^−5^, 5 × 10^−5^, 10^−4^, or 2 × 10^−4^ M) were added to the cells and then incubated for 24 h. After incubation, the cells were washed with PBS, and then the ROS production was determined by using DCFDA/H2DCFDA (Cellular ROS Assay Kit, Abcam^®^, Cambridge, UK). Briefly, 100 μL of ROS reagent were added (25 μM) to the cells and then incubated for 45 min at 37 °C. For the ROS measurements, the fluorescence intensity was detected using Synergy H1 Hybrid Multi-Mode Reader (BioTek US, Winooski, VT, USA) at the excitation/emission wavelengths of 485 nm/535 nm. 

### 2.7. Biological Trasmission Electron Microscopy (BIO-TEM)

B16F10 cells were seeded onto 24-well plates (2 × 10^4^ cells/well) and incubated o.n. The cells were then treated with BSA-Silver NPs (at 2 × 10^−3^ M as silver). After incubation for 1 h or 4 h, the cells were carefully washed 3 times with PBS and then detached by incubation with 0.25% trypsin-EDTA solution. The cells were then centrifuged at 2000 rpm for 3 min and, after removing the supernatant, a mixture solution of 2% formalin (Sigma-Aldrich, St. Louis, MO, USA) and 2.5% glutaraldehyde (Sigma-Aldrich) was added for fixation, and the cell pellets were incubated o.n. at 4 °C. The next day, after washing in 0.1 M phosphate buffer, the cells were fixed in 1% Osmium tetroxide (Sigma-Aldrich) for 1 h. After washing in 0.1 M phosphate buffer, cells were serially dehydrated using 50%, 60%, 70%, 80%, 90%, 95%, and, finally, 100% ethanol. After removal of ethanol, the cells were incubated in 100% propylene oxide for 10 min and then 2:1 mixture of propylene oxide and Epon A:B solution for 30 min. Epon A:B solution was prepared by freshly mixing Epon A and B solution (Epoxy-Embedding kit, Sigma-Aldrich). Epon A solution was composed of 5 mL of Epon-812 and 8 mL of DDSA (2-Dodecenylsuccinic anhydride) while Epon B solution contained 8 mL of Epon-812 and 7 mL of NMA (Methylnadic anhydride). Cells were incubated in 1:1 mixture of propylene oxide:Epon A:B for 1 h, followed by 1 h incubation in 1:2 mixture of propylene oxide:Epon A:B. After o.n. incubation in fresh Epon A:B solution, cell pellets were transferred to the embedding capsules (EMS, Hatfield, PA, USA) and incubated in Epon A:B with 1.5% DPM-30 (2,4,6-Tris(dimethylaminomethyl)phenol) for 12 h at 40 °C. Then, the cell blocks in capsules were hardened by incubation at 60 °C for 48 h. The sections from the embedded cell blocks were stained with uranyl acetate and lead citrate, and then put on a copper grid for the observation by TEM.

### 2.8. Tube Formation Assay

Extracellular matrix solution was added to pre-chilled 96 wells (50 μL per well) and incubated for 1 h in a cell incubator (37 °C, 5% CO_2_). After the preparation of the matrix gel, HUVEC, grown on 75T flasks harvested and re-suspended in Medium 200 (containing LSGS; ThermoFisher Scientific), were added to the wells (5 × 10^4^ cells/well) with the addition of PBS (negative control), BSA-Silver NPs (final silver concentration of 0, 10^−6^, 10^−5^ or 10^−4^ M) or anti-IGF-1R affibody (IAFF; 2 × 10^−5^ M; positive control). The recombinant SpyTag-IAFF protein was produced from *E. coli* in our laboratory [29]. After o.n. incubation, the tube formation of the cells was observed under a microscope, and the number of tubules was counted.

### 2.9. Cell Proliferation Assay

HUVEC (10^4^ cells/well), in a total volume of 100 mL of complete DMEM (with 10% FBS, 1% AA, and 1% PS), were plated to 96 well plates and then incubated o.n. in the cell incubator. After incubation, the medium was replaced with DMEM (with 1% FBS) and then VEGF (50 ng/mL), BSA-Silver NP (10^−4^ M), VEGF + BSA-Silver NP (50 ng/mL VEGF + 10^−4^ M BSA-Silver NPs) or VEGF + IAFF (50 ng/mL VEGF + 2 × 10^−5^ M IAFF) were added to the wells. The HUVEC were further incubated for 24 h, and then the relative cell viability was measured using the WST-1 assay (iNtRON Biotechnology).

### 2.10. Cell Migration Assay

HUVEC (3 × 10^4^ cells/well) were seeded onto a 96 well plate and, after o.n. incubation, a scratch wound was created by using a sterile plastic pipette tip. After removing the medium, the cells were washed with PBS twice and then incubated for 24 h with either VEGF (50 ng/mL), VEGF + BSA-Silver NP (50 ng/mL of VEGF + 10^−4^ M BSA-Silver NP), or VEGF + IAFF (50 ng/mL VEGF + 2 × 10^−5^ M IAFF). The migration distance of the cells was measured using the IncuCyte ZOOM (Essen Bioscience, Ann Arbor, MI, USA).

### 2.11. Photothermal Activity of BSA-Silver NPs

To evaluate the laser-induced photothermal activity of BSA-Silver NPs, the BSA-Silver NPs were prepared at varying concentrations (0–4 × 10^−3^ M as silver) in Eppendorf tubes and then irradiated with a laser (690 nm, 1.0 W, spot size, 5 × 8 mm^2^, MRL-III-690, Changchun New Industries Optoelectronics Tech Co. Ltd., Changchun, China) for 10 min. Furthermore, the particle samples (2.7 × 10^−3^ M as silver) were irradiated with a laser (690 nm) for 10 min at different laser powers (0.5–1.0 W). The temperature profiles of the samples were monitored using an E5 infrared camera (FLIR Systems, Boston, MA, USA). 

### 2.12. Evaluation of Cellular Phototoxic Effects by BSA-Silver NPs

PBS and BSA-Silver NPs (final 2.7 × 10^−3^ M as silver) were separately added to the B16F10 cells seeded on 96-well plates (5 × 10^3^ cells/well), and irradiated with a laser for 10 min to each well. Three different laser powers (0.8, 0.9, and 1.0 W) were chosen to acquire maximum temperatures of about 40, 45, and 50 °C, respectively, in the wells. Then, the cells were washed with PBS twice and further incubated for 48 h. The relative cell viability was evaluated using the WST-1 assay.

### 2.13. Statistical Analysis

All data are presented as means ± standard errors of the mean (SEM). One-way ANOVA and Tukey’s multiple comparison test (as the post hoc test) was used to determine the statistical significance. Any result yielding *p* < 0.05 was considered statistically significant.

## 3. Results and Discussions

### 3.1. Physical Characterization of BSA-Silver NPs

**Figure 1:**
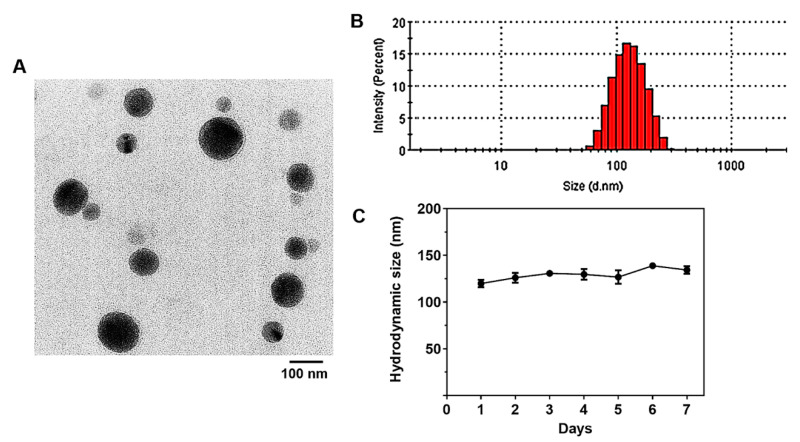
Physical characterizations of BSA-Silver NPs: (**A**) representative TEM images, (**B**) mean hydrodynamic size measured by dynamic light scattering, and (**C**) size stability. (BSA-Silver NPs: BSA-coated silver nanoparticles, TEM: transmission electron microscopy).

In this study, BSA-Silver NPs were synthesized by the reduction of AgNO_3_ in the presence of BSA. After the preparation of BSA-Silver NPs, their morphology and size were examined. In the TEM images, BSA-Silver NPs appeared spherical and well dispersed with the mean diameters of about 100 nm (Figure 1A). Through the light contrast in the NPs’ images, the silver NP core (darker) and the BSA coating (lighter) were recognized. According to the DLS data, the mean hydrodynamic size of the NPs was 121.2 (±43) nm (PDI: 0.095) (Figure 1B), while the zeta potential was −31.3 (±5.3) mV.

To date, different coating materials (e.g., citrate, polyvinylpyrrolidone, chitosan, poly (DL-Lactide-co-Glycolide), etc.) have been adopted for the synthesis of silver NPs [30,31,32]. As an NP coating material, albumin possesses unique advantageous properties such as high water solubility, stability, a long plasma circulation time (plasma half-live of human serum albumin: 19 days [33]), and avid binding ability to various substances (e.g., fatty acids, and small molecule drugs [34,35]). Most importantly, albumin can extend the plasma circulation time of the coated NPs by itself, while other coating materials usually require further PEGylation to ensure sufficient plasma residence. Previously, we reported that BSA-coated Silver NPs could reside in the bloodstream for about an hour without any other modification [36]. In addition, due to albumin’s propensity to bind with different types of drug molecules and materials (such as fatty acids), the BSA-coated NPs can serve as effective drug carriers [37]. Notably, in the present study, with the help of the BSA coating, BSA-Silver NPs could maintain their size for five days, which indicates their good stability (Figure 1C). 

**Figure 2:**
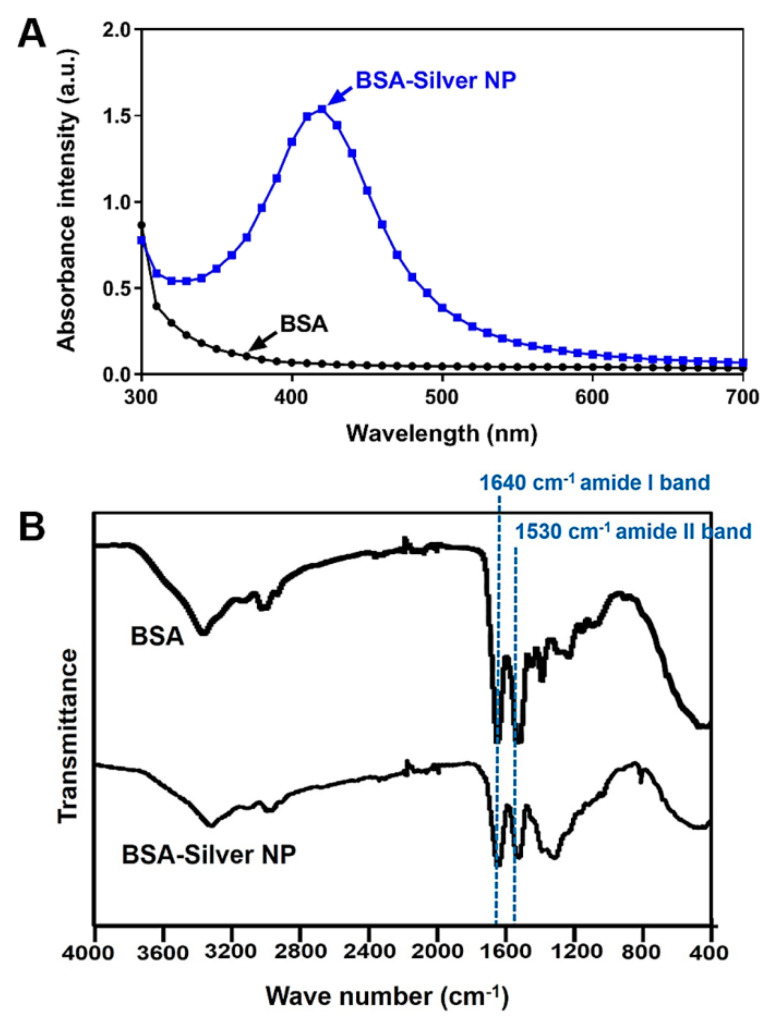
UV/VIS absorption and FT-IR spectra of BSA-Silver NPs: (**A**) UV-VIS absorption spectrum and (**B**) FT-IR spectrum. The representative surface plasmon resonance peak of silver NPs was observed at 420 nm from the BSA-Silver NPs’ UV-VIS spectrum. Additionally, in the FT-IR spectrum, the peaks at 1530 and 1640 cm^−^^1^ responsible for the amide bonds in the BSA verified the presence of the BSA coating. (BSA-Silver NPs: BSA-coated silver nanoparticles, UV/VIS: ultraviolet/visible).

The successful transformation of silver ions to silver NPs was further evidenced by the results of UV-VIS spectroscopy. As shown in Figure 2A, the single peak at 420 nm with a well-known characteristic band of silver NPs was observed from the spectrum of BSA-Silver NPs. On the other hand, the incorporation of the BSA into the NPs was confirmed by the FT-IR (Figure 2B). As can be seen in Figure 2B, two new distinct peaks were found from the spectrum of BSA-Silver NPs. These peaks at 1640 and 1530 cm^−1^ corresponded to the amide I and II bands generally found from the IR spectrum of proteins including the BSA [38]. Overall, through a series of physical characterizations, the successful synthesis of BSA-Silver NPs was confirmed.

### 3.2. Cytotoxicity and ROS Generation by BSA-Silver NPs

**Figure 3:**
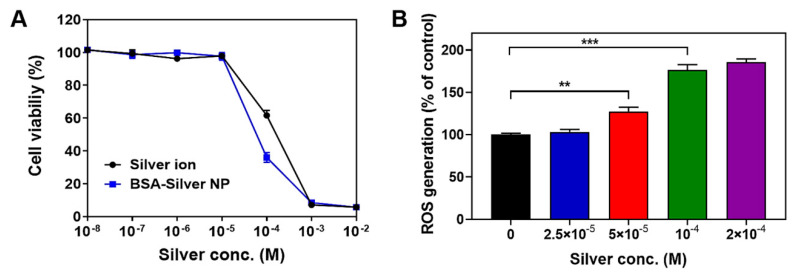
Cytocidal activity of BSA-Silver NPs: (**A**) cell viability profiles of B16F10 cells after treatment of either silver ions or BSA-Silver NPs. (**B**) Reactive oxygen species (ROS) generation assay results. The statistically significant differences in the cytotoxicity levels were compared by 1-way ANOVA (Tukey’s multiple comparison test as the post hoc test). ** *p* < 0.01, *** *p* < 0.001 vs. PBS control. (BSA-Silver NP: BSA-coated silver nanoparticles).

The cytotoxicity of silver has long been recognized as a potential concern for its use in clinical applications; however, in recent years, researchers have attempted to exploit this property to kill cancer cells [2,39]. To date, the anti-cancer effects of silver NPs have been reported for various cancer cell types, including glioblastoma, lung, ovarian, breast and melanoma cells [2]. To verify the cytocidal activity of BSA-Silver NPs, we performed a cell viability study (Figure 3A). Both the silver ions and the BSA-Silver NPs elicited marked cytotoxicity equivalent to silver ions on B6F10 melanoma cells. The half maximal inhibitory concentration (IC_50_) values for BSA-Silver NPs and the silver ions were 65.8 (±9.2) × 10^−6^ M and 120 (±14.4) × 10^−6^ M, respectively.

With regard to the mechanism for the anticancer properties, available evidence suggests that more than one process is involved in cell death. De Matteis et al. proposed that the silver NPs are up-taken by cells via endocytosis and, likely after degradation in the lysosomes, the released cytosolic silver ions may cause production of high intracellular ROS levels that could eventually induce DNA damage and mitochondria-involved apoptosis [40]. Gurunathan et al. reported that the silver nanoparticle cytotoxicity in the breast cancer cell line MDA-MB-231 was through a typical p53 dependent apoptotic pathway [41]. However, recently, autophagy was also suggested as a probable mechanism. This hypothesis was supported by Lin et al. who found that treatment of an autophagy inhibitor enhances the anticancer activity of silver NPs [42]. 

It has been well known that oxidative stress induced by a high level of ROS could cause damage to the plasma and mitochondrial membranes [40]. Specifically, for silver NPs, this ROS generation has frequently been reported as a major mechanism responsible for their cytotoxic activity [40,43]. Therefore, we evaluated the induction of ROS generation by BSA-Silver NPs on B16F10 cancer cells. The ROS generation assay results are shown in Figure 3B. BSA-Silver NPs indeed showed a dose-dependent increase on the cellular ROS level. Specifically, above 5 × 10^−5^ M (as silver), a significant increase of ROS generation was achieved (27, 76, and 86% increase for 5 × 10^−5^, 10^−4^, and 2 × 10^−4^ M (as silver), respectively). These results clearly evidence that BSA-Silver NPs could generate free radicals, which is consistent with previous reports [44].

### 3.3. BIO-TEM

**Figure 4:**
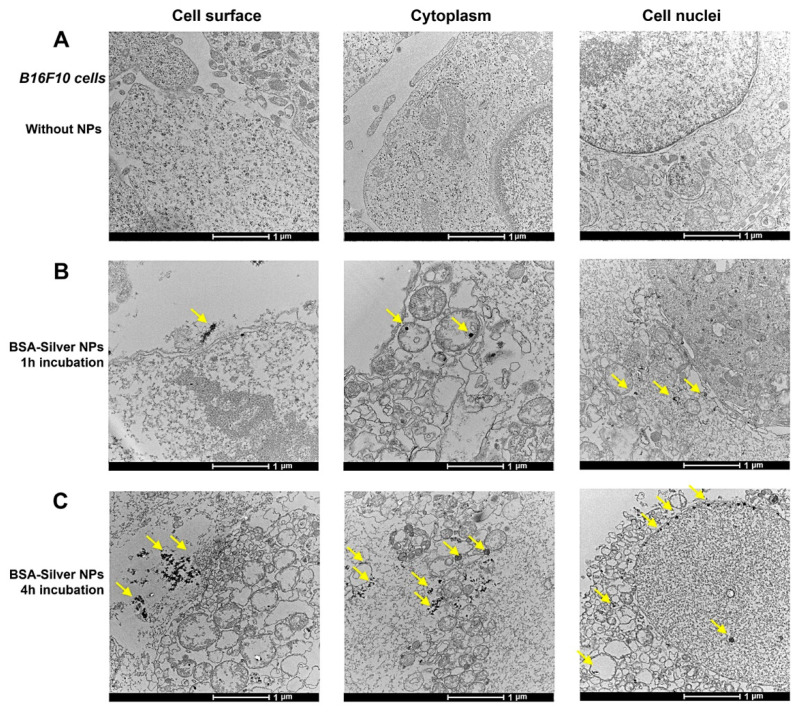
BIO-TEM examination of BSA-Silver NPs: (**A**) B16F10 cells without treatment; cells incubated with BSA-Silver NPs for (**B**) 1 h or (**C**) 4 h. BSA-Silver NPs are shown with the yellow arrows on the images. (BIO-TEM: biological transmission electron microscopy, BSA-Silver NP: BSA-coated silver nanoparticles).

The cellular internalization of silver NPs have been considered a pre-requisite for exerting their cytotoxic effects [45]. Hence, there have been many attempts to investigate the internalization pathways and their kinetics. Among the suggested various pathways, similar to other NPs, endocytosis has been considered a major route for silver NPs [45]. To verify the cellular behavior of BSA-Silver NPs, we performed a BIO-TEM of the B16F10 cells after incubation with BSA-Silver NPs. As shown in Figure 4, compared with the control cell (Figure 4A), increased numbers of internalized NPs could be observed inside the cells along the time course (Figure 4B,C). The cellular internalized BSA-Silver NPs generally maintained their original shapes and sizes. After 1 h incubation, some BSA-Silver NPs were observed near the outer cell membranes (Figure 4B-left), while others could be found in endocytic vesicles and the cytosol (Figure 4B-middle and right). These images evidenced that silver NPs could indeed enter the cells via endocytosis. At 4 h post-incubation, cytosolic accumulation of the NPs was more obviously identified and, furthermore, BSA-Silver NPs could be even found inside the nucleus (Figure 4C). These results were partly consistent with Wu et al., who reported size-dependent cellular internalization of citrate-coated silver NPs in B16 melanoma cells via endocytosis [45]. However, differing from their report, interestingly, nuclear accumulation could be observed within just 4 h of incubation in spite of the large size (121 nm in diameter) of BSA-Silver NPs. To this regard, previous reports suggested that the cellular uptake efficiency and subcellular distribution of the NPs could be dependent upon their physicochemical properties [46]. Thus, this discrepancy may be explained by the difference in coating materials, but further studies would be required to clarify this issue.

### 3.4. Inhibition of VEGF-Induced Angiogenesis by BSA-Silver NPs

Angiogenesis sustains tumor growth by expanding the blood supply and providing essential nutrients to tumor cells [47]. It was even reported that, without sufficient blood supply, a tumor may not be able to increase beyond 2–3 mm in diameter [48]. Apart from tumor growth, angiogenesis also endows tumor cells with a favorable environment to spread out through the bloodstream, which leads to metastasis [49]. Due to this significant contribution to the development of tumors, angiogenesis has been one of the major targets of cancer chemotherapy. Generally, angiogenesis is initiated by the release of angiogenic stimulators such as growth factors (e.g., VEGF) from tumor cells [47,50]. By the synchronized work of growth factors and proteolytic enzymes, new blood vessels would grow and form new vascular networks. As endothelial tube formation, proliferation, and migration are the key steps for angiogenesis, and the inhibitory effects of BSA-Silver NPs on each of these steps were tested on HUVEC.

**Figure 5:**
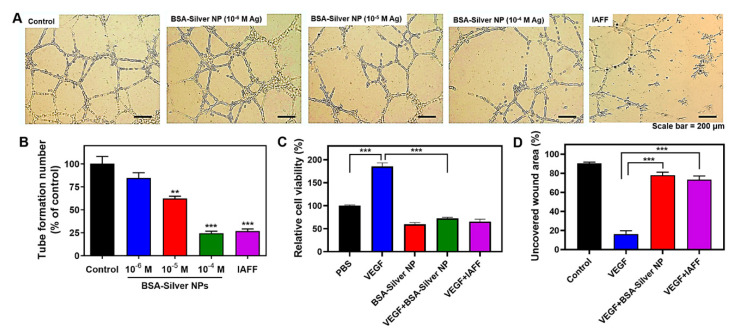
Anti-angiogenic activity of BSA-Silver NPs. (**A**) Representative images of HUVEC treated with BSA-Silver NPs or IAFF and (**B**) tubule numbers in the tube formation assay. (**C**) Cell proliferation and (**D**) cell migration assay results. The statistically significant differences were compared by one-way ANOVA (Tukey’s multiple comparison test as the post hoc test). ** *p* < 0.01, *** *p* < 0.001 vs. PBS control. (BSA-Silver NP: BSA-coated silver nanoparticles, HUVEC: human umbilical vein endothelial cells, IAFF: anti-insulin-like growth factor 1 receptor affibody).

To evaluate the inhibitory effects of BSA-Silver NPs on tube formation, a typical matrix gel-based tube formation assay was performed with HUVEC. The tube formation assay results are shown in Figure 5A,B. After incubation in the conditioned medium (Medium 200 with LSGS), the HUVEC formed networks of many tubules. However, when compared to the negative control group, the cells treated with BSA-Silver NPs showed dose-dependent inhibition of tube formation. The relative counts of the formed tubules were 84.8, 62.4, and 24.6% with the treatment of BSA-Silver NPs at 10^−6^, 10^−5^, and 10^−4^ M, respectively. At 10^−4^ M, BSA-Silver NPs yielded equivalent inhibitory effects to those of the positive control IAFF (2 × 10^−5^ M). These results suggest that BSA-Silver NPs could indeed block the formation of new blood vessels. Regarding the underlying cellular mechanism, the Eom research group suggested that these inhibitory effects may be related to the inactivation of the PI3K/Akt pathway by silver NPs [14,51].

The effects of BSA-Silver NPs on the proliferation of endothelial cells were also tested on VEGF-induced HUVEC. The assay results are shown in Figure 5C. While VEGF induced a significant increase in the number of viable cells, BSA-Silver NPs yielded a marked reduction, such as IAFF. The effects of BSA-Silver NPs on the mobility of endothelial cells were also evaluated by a typical wound-healing assay. As shown in Figure 5D, the VEGF could have induced endothelial cell migration to nearly close the entire wound area (84%) after 24 h incubation. However, in sharp contrast from the wells treated with VEGF + BSA-Silver NPs and VEGF + IAFF, a large area of the wound remained uncovered (78% and 73%, respectively) with cells, suggesting that BSA-Silver NPs could effectively block the migration of VEGF-stimulated endothelial cells. These findings are consistent with results reported by Gurunathan et al. [14]. Taken together, our results revealed that BSA-Silver NPs are capable of inhibiting angiogenesis. 

### 3.5. Photothermal Effects by BSA-Silver NPs

**Figure 6:**
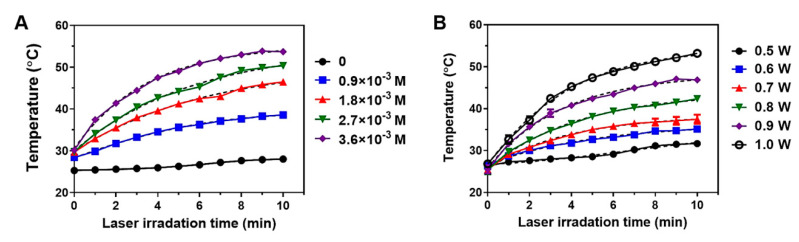
Photothermal activity of BSA-Silver NPs. Temperature profiles of BSA-Silver NPs after laser irradiation at (**A**) 1 W with varying sample concentrations (0–3.6 × 10^−3^ M as silver) and (**B**) at 2.7 × 10^−3^ M with varying laser powers (0.5–1.0 W). Dotted lines are showing the non-linear regression fitted curves of the original data curves. (BSA-Silver NPs: BSA-coated silver nanoparticles).

Recently, PTT has emerged as an attractive alternative to conventional modes of cancer therapies [16]. This is so because, by focally irradiating an external laser to the tumor site, spatially selective and minimally invasive treatment could be accomplished. The therapeutic effects of PTT heavily rely on the light-to-heat conversion efficiency of the photothermal agents, as well as on the accessibility of the tumor tissue to the laser [36]. Therefore, to date, various NPs composed of different materials, including silver NPs, have been explored in the PTT research [52]. In the present study, we evaluated the photothermal activity of BSA-Silver NPs. Figure 6 shows the light-to-heat conversion capacity of BSA-Silver NPs in different NP concentrations and laser powers. As seen in Figure 6A,B, with the irradiation of the laser to BSA-Silver NPs, the suspension temperature increased and gradually reached a plateau at about 10 min post-irradiation. Based on the temperature profiles, we found that BSA-Silver NPs were stable under the laser. Furthermore, increasing the NP concentration yielded a higher temperature rise. By applying the nonlinear regression model (with variable slope) to the curves, the estimated maximum temperatures were 28.4, 39.7, 50.7, 54.8, and 55.4 °C at 0, 0.9 × 10^−3^, 1.8 × 10^−3^, 2.7 × 10^−3^, and 3.6 × 10^−3^ M of BSA-Silver NP concentrations (as silver), respectively. As shown in Figure 6B, the photothermal activity of BSA-Silver NPs also positively correlated with the laser power (+8.1, +10.0, +12.9, +18.4, +22.7, and +27.4 °C at 0.5, 0.6, 0.7, 0.8, 0.9, and 1.0 W, respectively). These findings are quite encouraging, because, at the applied laser wavelength (690 nm), light absorption may have been very low (Figure 2A). However, the results still showed a significant temperature rise in millimolar concentrations. In this regard, Thompson et al. suggested an effective way to improve the PTT effects of silver NPs was by synthesizing silver NPs optimized for absorbing the light in the NIR range (maximum absorption peak at about 800 nm) [25].

**Figure 7:**
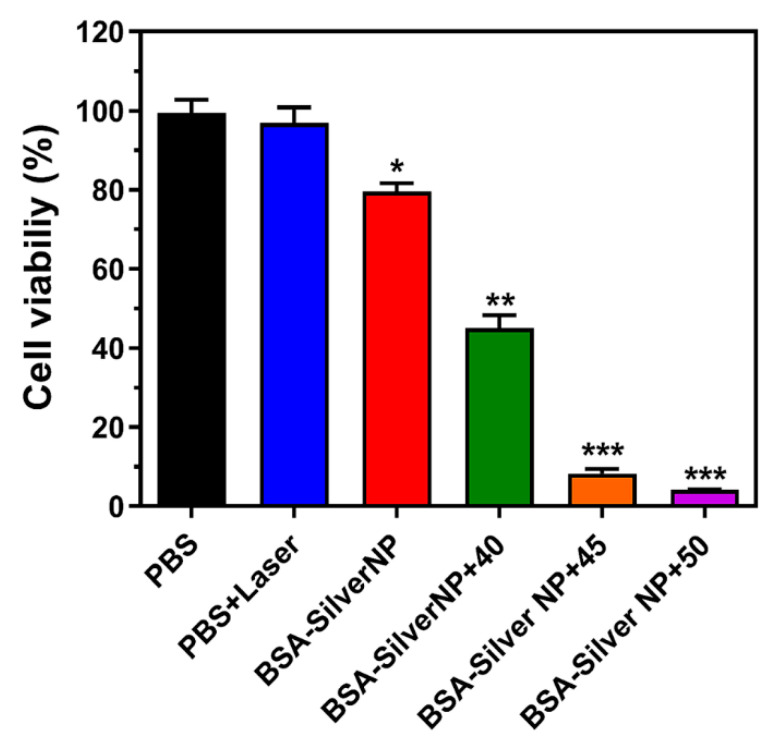
Anti-cancer activity of BSA-Silver NPs with photothermal treatment. Cytotoxic effects of laser irradiation with or without treatment of BSA-Silver NPs were evaluated on B16F10 cells. Laser power was set to achieve maximum mean temperatures of 40, 45, or 50 °C. The statistically significant differences in the cytotoxicity levels were compared by 1-way ANOVA (Tukey’s multiple comparison test as the post hoc test). * *p* < 0.05, ** *p* < 0.01, *** *p* < 0.001 vs. PBS control. (BSA-Silver NPs: BSA-coated silver nanoparticles).

In the next step, the anti-cancer activity of BSA-Silver NPs with thermal treatment was evaluated on B16F10 cells. As shown in Figure 7, the cells treated with a laser (at 1 W) without BSA-Silver NPs showed a limited temperature rise (less than 40 °C) and did not show apparent signs of cytotoxicity. However, in comparison, the cells treated with both BSA-Silver NPs and laser exhibited a significant decrease in viability. Specifically, at temperatures above 45 °C, almost complete cell death was achieved (mean cell viability: 99.4, 8.2, and 4.2% for PBS, BSA-Silver NP + 45, and BSA-Silver NP + 50, respectively). These results are in good accordance with previous reports [25] and clearly demonstrate the potential applicability of BSA-Silver NPs for PTT in the treatment of skin cancer.

Different mechanisms have been suggested for cancer cell death by PTT. Among these, two major pathways include necrosis and apoptosis. While apoptosis is a programmed cell death, necrosis is a pre-mature “less clean” type of cell death caused by cell damage [53]. Due to the hyperthermic nature of PTT, the necrotic pathway has been considered to be the more dominant process for PTT-induced cell death [53]. However, the results of available reports are controversial and, recently, even novel mechanisms, such as necroptosis, a programmed-type necrosis, have been proposed [54]. Pattani et al. reported that the apoptosis to necrosis ratio could vary depending upon the intracellular location of the NPs, which could be affected by incubation time and NP size [55]. With an increased incubation time before the laser irradiation, higher apoptosis ratio was reported. This finding was explained by a higher fraction of internalized NPs [55]. According to Wu et al., cellular uptake and localization profiles of the silver NPs could also be affected by particle size [45]. Considering the interrelated, but different tumoricidal mechanisms between silver NPs and PTT, a combination therapy may provide a synergic (or additive) effect in killing cancer cells. Moreover, when loading chemotherapeutics onto silver NPs, the increased cytosolic release of the drugs by PTT may further enhance therapeutic effects.

## 4. Conclusions

In the present study, BSA-Silver NPs were successfully synthesized and characterized with the focus on their anti-cancer activity specifically in skin cancer treatment. BSA-Silver NPs, prepared by a single-step reduction process, showed good stability and appropriate size distribution for pharmaceutical applications. Cellular analyses revealed that BSA-Silver NPs could internalize melanoma cells and kill via inducing ROS, while, on the other hand, BSA-Silver NPs were found to be potentially active in inhibiting angiogenesis. Furthermore, BSA-Silver NPs were found to possess a great light-to-heat conversion ability that could be potentially exploited for photothermal cancer therapy. Taken together, our results suggest that BSA-Silver NPs could serve as potent multimodal therapeutic agents for the effective treatment of skin cancer.

## Data Availability

Not applicable.

## References

[1] Foulkes R., Ali Asgari M., Curtis A., Hoskins C. (2019). Silver-nanoparticle-mediated therapies in the treatment of pancreatic cancer. ACS Appl. Nano Mater..

[2] Zhang X.-F., Liu Z.-G., Shen W., Gurunathan S. (2016). Silver nanoparticles: Synthesis, characterization, properties, applications, and therapeutic approaches. Int. J. Mol. Sci..

[3] Sanpui P., Chattopadhyay A., Ghosh S.S. (2011). Induction of apoptosis in cancer cells at low silver nanoparticle concentrations using chitosan nanocarrier. ACS Appl. Mater. Interfaces.

[4] Ma W., Jing L., Valladares A., Mehta S.L., Wang Z., Li P.A., Bang J. (2015). Silver nanoparticle exposure induced mitochondrial stress, caspase-3 activation and cell death: Amelioration by sodium selenite. Int. J. Biol. Sci..

[5] Guo D., Zhu L., Huang Z., Zhou H., Ge Y., Ma W., Wu J., Zhang X., Zhou X., Zhang Y. (2013). Anti-leukemia activity of PVP-coated silver nanoparticles via generation of reactive oxygen species and release of silver ions. Biomaterials.

[6] Yang T., Yao Q., Cao F., Liu Q., Liu B., Wang X.-H. (2016). Silver nanoparticles inhibit the function of hypoxia-inducible factor-1 and target genes: Insight into the cytotoxicity and antiangiogenesis. Int. J. Nanomed..

[7] Zhang X.-F., Shen W., Gurunathan S. (2016). Silver nanoparticle-mediated cellular responses in various cell lines: An in vitro model. Int. J. Mol. Sci..

[8] Huy T.Q., Huyen P., Le A.-T., Tonezzer M. (2020). Recent advances of silver nanoparticles in cancer diagnosis and treatment. Anti-Cancer Agents Med. Chem..

[9] Durán N., Silveira C.P., Durán M., Martinez D.S.T. (2015). Silver nanoparticle protein corona and toxicity: A mini-review. J. Nanobiotechnol..

[10] Galindo T.G.P., Chai Y., Tagaya M. (2019). Hydroxyapatite nanoparticle coating on polymer for constructing effective biointeractive interfaces. J. Nanomater..

[11] Battaglia L., Ugazio E. (2019). Lipid nano-and microparticles: An overview of patent-related research. J. Nanomater..

[12] Kerbel R.S. (2000). Tumor angiogenesis: Past, present and the near future. Carcinogenesis.

[13] Hashemi Goradel N., Ghiyami-Hour F., Jahangiri S., Negahdari B., Sahebkar A., Masoudifar A., Mirzaei H. (2018). Nanoparticles as new tools for inhibition of cancer angiogenesis. J. Cell. Physiol..

[14] Gurunathan S., Lee K.-J., Kalishwaralal K., Sheikpranbabu S., Vaidyanathan R., Eom S.H. (2009). Antiangiogenic properties of silver nanoparticles. Biomaterials.

[15] Arvizo R.R., Rana S., Miranda O.R., Bhattacharya R., Rotello V.M., Mukherjee P. (2011). Mechanism of anti-angiogenic property of gold nanoparticles: Role of nanoparticle size and surface charge. Nanomedicine.

[16] Zou L., Wang H., He B., Zeng L., Tan T., Cao H., He X., Zhang Z., Guo S., Li Y. (2016). Current approaches of photothermal therapy in treating cancer metastasis with nanotherapeutics. Theranostics.

[17] Chatterjee D.K., Diagaradjane P., Krishnan S. (2011). Nanoparticle-mediated hyperthermia in cancer therapy. Ther. Deliv..

[18] Nielsen O.S., Horsman M., Overgaard J. (2001). A future for hyperthermia in cancer treatment?. Eur. J. Cancer.

[19] Kampinga H., Dikomey E. (2001). Hyperthermic radiosensitization: Mode of action and clinical relevance. Int. J. Radiat. Biol..

[20] Prasad N., Rathinasamy K., Panda D., Bahadur D. (2007). Mechanism of cell death induced by magnetic hyperthermia with nanoparticles of γ-Mn x Fe 2 − x O 3 synthesized by a single step process. J. Mater. Chem..

[21] Vines J.B., Yoon J.-H., Ryu N.-E., Lim D.-J., Park H. (2019). Gold Nanoparticles for Photothermal Cancer Therapy. Front. Chem..

[22] Jain P.K., Huang X., El-Sayed I.H., El-Sayed M.A. (2008). Noble metals on the nanoscale: Optical and photothermal properties and some applications in imaging, sensing, biology, and medicine. Acc. Chem. Res..

[23] Wei W., Zhang X., Zhang S., Wei G., Su Z. (2019). Biomedical and bioactive engineered nanomaterials for targeted tumor photothermal therapy: A review. Mater. Sci. Eng. C.

[24] Liu Y., Crawford B.M., Vo-Dinh T. (2018). Gold nanoparticles-mediated photothermal therapy and immunotherapy. Immunotherapy.

[25] Thompson E.A., Graham E., MacNeill C.M., Young M., Donati G., Wailes E.M., Jones B.T., Levi-Polyachenko N.H. (2014). Differential response of MCF7, MDA-MB-231, and MCF 10A cells to hyperthermia, silver nanoparticles and silver nanoparticle-induced photothermal therapy. Int. J. Hyperth..

[26] Boca S.C., Potara M., Gabudean A.-M., Juhem A., Baldeck P.L., Astilean S. (2011). Chitosan-coated triangular silver nanoparticles as a novel class of biocompatible, highly effective photothermal transducers for in vitro cancer cell therapy. Cancer Lett..

[27] Liu Y., Li F., Guo Z., Xiao Y., Zhang Y., Sun X., Zhe T., Cao Y., Wang L., Lu Q. (2020). Silver nanoparticle-embedded hydrogel as a photothermal platform for combating bacterial infections. Chem. Eng. J..

[28] Wang N., Hu B., Chen M.-L., Wang J.-H. (2015). Polyethylenimine mediated silver nanoparticle-decorated magnetic graphene as a promising photothermal antibacterial agent. Nanotechnology.

[29] Ham S., Min K.A., Yang J.W., Shin M.C. (2017). Fusion of gelonin and anti-insulin-like growth factor-1 receptor (IGF-1R) affibody for enhanced brain cancer therapy. Arch. Pharm. Res..

[30] Chaubey N., Sahoo A.K., Chattopadhyay A., Ghosh S.S. (2014). Silver nanoparticle loaded PLGA composite nanoparticles for improving therapeutic efficacy of recombinant IFNgamma by targeting the cell surface. Biomater. Sci..

[31] Wang H., Qiao X., Chen J., Wang X., Ding S. (2005). Mechanisms of PVP in the preparation of silver nanoparticles. Mater. Chem. Phys..

[32] Murugadoss A., Chattopadhyay A. (2007). A ‘green’chitosan–silver nanoparticle composite as a heterogeneous as well as micro-heterogeneous catalyst. Nanotechnology.

[33] Caraceni P., Tufoni M., Bonavita M.E. (2013). Clinical use of albumin. Blood Transfus..

[34] Koch-Weser J., Sellers E.M. (1976). Binding of drugs to serum albumin. N. Engl. J. Med..

[35] Kurtzhals P., Havelund S., Jonassen I., Markussen J. (1997). Effect of fatty acids and selected drugs on the albumin binding of a long-acting, acylated insulin analogue. J. Pharm. Sci..

[36] Park T., Lee S., Amatya R., Cheong H., Moon C., Kwak H.D., Min K.A., Shin M.C. (2020). ICG-loaded PEGylated BSA-silver nanoparticles for effective photothermal cancer therapy. Int. J. Nanomed..

[37] Hong S., Choi D.W., Kim H.N., Park C.G., Lee W., Park H.H. (2020). Protein-based nanoparticles as drug delivery systems. Pharmaceutics.

[38] Grdadolnik J., Maréchal Y. (2001). Bovine serum albumin observed by infrared spectrometry. I. Methodology, structural investigation, and water uptake. Biopolymers.

[39] Reidy B., Haase A., Luch A., Dawson K.A., Lynch I. (2013). Mechanisms of silver nanoparticle release, transformation and toxicity: A critical review of current knowledge and recommendations for future studies and applications. Materials.

[40] De Matteis V., Malvindi M.A., Galeone A., Brunetti V., De Luca E., Kote S., Kshirsagar P., Sabella S., Bardi G., Pompa P.P. (2015). Negligible particle-specific toxicity mechanism of silver nanoparticles: The role of Ag+ ion release in the cytosol. Nanomed. Nanotechnol. Biol. Med..

[41] Gurunathan S., Park J.H., Han J.W., Kim J.H. (2015). Comparative assessment of the apoptotic potential of silver nanoparticles synthesized by Bacillus tequilensis and Calocybe indica in MDA-MB-231 human breast cancer cells: Targeting p53 for anticancer therapy. Int. J. Nanomed..

[42] Lin J., Huang Z., Wu H., Zhou W., Jin P., Wei P., Zhang Y., Zheng F., Zhang J., Xu J. (2014). Inhibition of autophagy enhances the anticancer activity of silver nanoparticles. Autophagy.

[43] Gopinath P., Gogoi S.K., Chattopadhyay A., Ghosh S.S. (2008). Implications of silver nanoparticle induced cell apoptosis for in vitro gene therapy. Nanotechnology.

[44] Awasthi K.K., Awasthi A., Kumar N., Roy P., Awasthi K., John P. (2013). Silver nanoparticle induced cytotoxicity, oxidative stress, and DNA damage in CHO cells. J. Nanopart. Res..

[45] Wu M., Guo H., Liu L., Liu Y., Xie L. (2019). Size-dependent cellular uptake and localization profiles of silver nanoparticles. Int. J. Nanomed..

[46] Chithrani B.D., Chan W.C. (2007). Elucidating the mechanism of cellular uptake and removal of protein-coated gold nanoparticles of different sizes and shapes. Nano Lett..

[47] Bagri A., Kouros-Mehr H., Leong K.G., Plowman G.D. (2010). Use of anti-VEGF adjuvant therapy in cancer: Challenges and rationale. Trends Mol. Med..

[48] Nishida N., Yano H., Nishida T., Kamura T., Kojiro M. (2006). Angiogenesis in cancer. Vasc. Health Risk Manag..

[49] Atlihan-Gundogdu E., Ilem-Ozdemir D., Ekinci M., Ozgenc E., Demir E.S., Sánchez-Dengra B., González-Alvárez I. (2020). Recent developments in cancer therapy and diagnosis. J. Pharm. Investig..

[50] Mirhadi E., Nassirli H., Malaekeh-Nikouei B. (2020). An updated review on therapeutic effects of nanoparticle-based formulations of saffron components (safranal, crocin, and crocetin). J. Pharm. Investig..

[51] Kalishwaralal K., Banumathi E., Pandian S.R.K., Deepak V., Muniyandi J., Eom S.H., Gurunathan S. (2009). Silver nanoparticles inhibit VEGF induced cell proliferation and migration in bovine retinal endothelial cells. Colloids Surf. B Biointerfaces.

[52] Graham E.G., Macneill C.M., Levi-Polyachenko N.H. (2013). Review of metal, carbon and polymer nanoparticles for infrared photothermal therapy. Nano Life.

[53] Melamed J.R., Edelstein R.S., Day E.S. (2015). Elucidating the fundamental mechanisms of cell death triggered by photothermal therapy. ACS Nano.

[54] Zhang Y., Zhan X., Xiong J., Peng S., Huang W., Joshi R., Cai Y., Liu Y., Li R., Yuan K. (2018). Temperature-dependent cell death patterns induced by functionalized gold nanoparticle photothermal therapy in melanoma cells. Sci. Rep..

[55] Pattani V.P., Shah J., Atalis A., Sharma A., Tunnell J.W. (2015). Role of apoptosis and necrosis in cell death induced by nanoparticle-mediated photothermal therapy. J. Nanopart. Res..

